# District-Level Estimates of Child Mortality in Uttar Pradesh, India

**DOI:** 10.7759/cureus.103889

**Published:** 2026-02-19

**Authors:** Ajay Pandey, Richa Sharma

**Affiliations:** 1 Population Research Center, University of Lucknow, Lucknow, IND; 2 Research, National Institute of Labour Economics Research and Development, New Delhi, IND

**Keywords:** aspirational districts, child mortality, child mortality rate, neonatal mortality, u5mr, uttar pradesh

## Abstract

The study examines district-wise variations in child mortality across Uttar Pradesh using the National Family Health Survey (NFHS)-5 dataset for Uttar Pradesh, by estimating the neonatal mortality rate (NMR), post-neonatal mortality rate (PNMR), child mortality rate (CMR), and under-five mortality rate (U5MR). Findings suggest that Uttar Pradesh continues to exhibit one of India's highest child mortality burdens, as the estimates are above the national average. Neonatal mortality, averaging about 36 deaths per 1,000 live births, remains the largest contributor to child deaths. Several central and western districts, such as Amethi, Mainpuri, and Shahjahanpur, record NMRs above 60, while some eastern districts like Ballia and Deoria have achieved rates below 12, nearing the 2030 Sustainable Development Goals (SDG). Post-neonatal mortality averages around 15 per 1,000, with high levels in Agra, Hardoi, and Kheri, indicating gaps in infant care and immunization coverage. In contrast, districts such as Kushinagar and Muzaffarnagar have PNMRs under 3, reflecting effective community-level health programs. Child mortality (ages 1-4) has declined significantly, with a state average near 10 per 1,000. Most districts report single-digit CMRs, but outliers like Fatehpur and Allahabad remain high, suggesting persistent issues related to nutrition and healthcare quality. The composite U5MR stands at nearly 60 deaths per 1,000 live births, still more than twice the SDG target. Extremes range from 111 in Amethi and 101 in Shahjahanpur to below 25 in Jaunpur, Muzaffarnagar, and Deoria. This divergence highlights both successful interventions and critical shortfalls within the state. The eight aspirational districts Bahraich, Balrampur, Chandauli, Chitrakoot, Fatehpur, Shravasti, Siddharthnagar, and Sonbhadra perform marginally better than the state average (U5MR 56.5 vs. 59.9), suggesting positive effects of targeted development initiatives. However, internal variation persists, with Siddharthnagar performing exceptionally well and Fatehpur lagging behind.

Overall, Uttar Pradesh's child mortality has declined but remains uneven. Sustained improvements in neonatal care, maternal health, nutrition, and equitable healthcare delivery, especially in high-mortality districts, are crucial for meeting national and SDG by 2030.

## Introduction

Child mortality is a critical indicator of a region's health infrastructure, maternal care, and socioeconomic conditions. India contributes significantly to the global child mortality burden, with Uttar Pradesh (UP) accounting for the highest number of neonatal and child deaths among Indian states [1]. Despite numerous national health interventions such as the National Health Mission (NHM) and Janani Suraksha Yojana (JSY), the neonatal and child mortality rate in UP remains persistently high [2].

Estimating child mortality indicators at the district level is vital for targeted policy planning and resource allocation. However, due to limitations in the availability of disaggregated and reliable data, district-level estimates are often underdeveloped. The National Family Health Survey (NFHS) and Sample Registration System (SRS) provide state-level data, but district-specific analysis is required to identify high-risk areas. Advanced statistical modeling and geospatial analysis are emerging as promising methods to overcome data limitations and provide granular insights into regional disparities [3].

This study aims to provide updated and statistically robust district-level estimates of child mortality in UP. The findings will help policymakers, public health officials, and non-governmental organizations (NGOs) in designing area-specific interventions to reduce neonatal deaths and achieve Sustainable Development Goal (SGD) 3.2, ending preventable deaths of newborns by 2030.

Numerous studies have explored the determinants and spatial distribution of neonatal and child mortality in India, highlighting stark inter- and intra-state disparities. According to Ram et al. [4], socioeconomic factors such as maternal education, household income, and access to antenatal care are strongly linked to neonatal survival.

UP has been a focal point in neonatal and child mortality research due to its population size and health challenges. Singh et al. [5] observed that NMR in rural UP is significantly higher than in urban areas, largely due to inadequate neonatal and childcare facilities and delayed healthcare-seeking behavior. Moreover, Singh and Moksha [6] emphasized that traditional practices, lack of skilled birth attendance, and nutritional deficiencies contribute substantially to neonatal deaths in remote districts.

Recent efforts to map mortality at the district level have adopted model-based approaches. For instance, Dandona et al. [7] applied small area estimation techniques to generate district-wise child mortality data, highlighting hotspots in eastern UP. Similarly, Fadel et al. [8] used geospatial modeling to identify regional clusters of neonatal deaths across northern India. These approaches underscore the potential of integrating household surveys, census data, and statistical models for granular insights.

The NFHS-5 and SRS reports are the most frequently used data sources but often lack the granularity needed for micro-level planning [2]. Several scholars advocate for combining multiple data sources, including the Civil Registration and Vital Statistics (CRVS), the Health Management Information System (HMIS), and the National Sample Surveys, to improve precision [9,10].

Despite increased data availability, there remains a gap in district-wise estimates of neonatal and child mortality specific to UP. Most studies focus on state-level trends or use older datasets. This research intends to bridge that gap by generating updated, district-specific estimates using modern statistical techniques and secondary datasets.

## Materials and methods

This study utilized birth history data from the NFHS-5 [11] for UP to estimate neonatal mortality rates at the district level. The NFHS-5 [11] provides comprehensive individual and household-level data on fertility, mortality, and maternal and child health indicators, making it a reliable source for such analysis. Specifically, the study extracted and analyzed children's birth history files for all births occurring in the five years preceding the survey. To ensure methodological consistency and accuracy, the analysis employed the STATA do-file provided by the Demographic and Health Survey (DHS) Program, which is specifically designed for calculating mortality indicators such as neonatal, infant, and under-five mortality (DHS-Indicators-Stata/Chap08_CM/CM_CHILD.do at master · DHSProgram/DHS-Indicators-Stata · GitHub). The estimates generated were disaggregated at the district level to uncover spatial variations within the state of UP.

## Results

Table 1 provides the district-wise estimates of child mortality indicators for UP. Each indicator is discussed in detail below.

**Table 1:** Estimates of child mortality indicators in Uttar Pradesh, India *Bahraich, Balrampur, Chandauli, Chitrakoot, Fatehpur, Shravasti, Siddharthnagar, and Sonbhadra NMR: neonatal mortality rate per 1000 live births (deaths occurring in the first month of life); IMR: infant mortality rate per 1000 live births (deaths occurring in the first year of life); PNMR: post-neonatal mortality (deaths occurring after one month of life and less than 12 months of life); CMR: child mortality rate per 1000 live births (infant deaths occurring in between one and five years); U5MR: under-five mortality rate per 1000 live births (infant deaths below age five) Sustainable Development Goal target for 2030: NMR ≤12 deaths per 1000 live births and U5MR ≤25 deaths per 1000 live births Reference: National Family Health Survey-5 (2019-2021) [11]

	District	NMR	PNMR	IMR	CMR	U5MR
1	Saharanpur	39.71	13.51	53.23	9.09	61.84
2	Bijnor	35.76	12.93	48.69	8.76	57.02
3	Rampur	48.20	19.67	67.88	11.86	78.93
4	Jyotiba Phule Nagar	36.14	17.31	53.46	8.61	61.61
5	Meerut	33.27	22.29	55.55	11.81	66.70
6	Baghpat	31.52	11.73	43.25	11.17	53.93
7	Gautam Buddha Nagar	39.63	11.32	50.95	2.25	53.09
8	Bulandshahr	45.22	20.13	65.36	15.41	79.76
9	Aligarh	41.20	16.73	57.93	3.35	61.08
10	Mahamaya Nagar	33.50	24.56	58.06	10.58	68.03
11	Mathura	35.14	20.39	55.53	3.80	59.12
12	Agra	31.71	32.41	64.11	8.77	72.32
13	Firozabad	51.36	21.96	73.32	7.73	80.48
14	Mainpuri	63.66	18.55	82.21	14.42	95.45
15	Bareilly	38.45	13.40	51.85	11.21	62.48
16	Pilibhit	23.07	13.64	36.71	16.75	52.85
17	Shahjahanpur	63.40	24.65	88.06	14.06	100.88
18	Kheri	48.75	25.31	74.07	17.99	90.72
19	Sitapur	60.64	20.42	81.06	16.20	95.95
20	Hardoi	57.24	26.65	83.89	9.90	92.96
21	Unnao	43.63	17.32	60.95	8.98	69.38
22	Lucknow	15.85	7.29	23.13	8.05	31.00
23	Farrukhabad	51.35	11.82	63.17	16.95	79.05
24	Kannauj	42.94	22.22	65.16	18.98	82.90
25	Etawah	39.34	19.52	58.86	11.83	69.99
26	Auraiya	34.00	21.99	55.99	12.52	67.81
27	Kanpur Dehat	41.51	14.32	55.83	13.54	68.61
28	Kanpur Nagar	28.78	14.03	42.81	11.34	53.66
29	Jalaun	25.00	9.54	34.55	3.04	37.48
30	Jhansi	20.10	19.23	39.33	8.87	47.85
31	Lalitpur	44.79	16.23	61.02	7.02	67.61
32	Hamirpur	34.89	12.61	47.50	0.00	47.50
33	Mahoba	31.30	13.04	44.34	11.23	55.07
34	Banda	38.91	19.92	58.83	11.50	69.66
35	Chitrakoot	31.73	10.83	42.56	6.77	49.04
36	Fatehpur	30.30	21.32	51.63	29.06	79.19
37	Pratapgarh	36.23	8.90	45.13	13.27	57.80
38	Kaushambi	30.06	14.81	44.87	16.77	60.89
39	Allahabad	37.68	15.26	52.94	24.91	76.53
40	Bara Banki	48.18	6.84	55.02	14.42	68.64
41	Faizabad	19.63	9.06	28.69	6.61	35.11
42	Ambedkar Nagar	20.77	12.83	33.60	9.75	43.02
43	Bahraich	41.92	18.68	60.60	8.69	68.77
44	Shrawasti	26.40	14.90	41.29	16.51	57.12
45	Balrampur	43.52	16.73	60.25	5.14	65.08
46	Gonda	18.69	15.85	34.55	11.08	45.24
47	Siddharthnagar	13.49	3.33	16.82	3.77	20.52
48	Basti	23.53	8.47	32.00	1.83	33.78
49	Sant Kabir Nagar	20.68	7.33	28.02	6.27	34.11
50	Mahrajganj	23.02	6.85	29.87	14.73	44.16
51	Gorakhpur	32.27	7.10	39.37	7.66	46.73
52	Kushinagar	22.08	1.91	23.98	5.90	29.75
53	Deoria	11.28	4.94	16.22	2.55	18.73
54	Azamgarh	26.11	20.16	46.27	2.03	48.21
55	Mau	49.15	3.04	52.20	4.91	56.85
56	Ballia	9.80	11.45	21.25	5.77	26.90
57	Jaunpur	11.77	2.92	14.70	2.76	17.41
58	Ghazipur	13.27	7.17	20.44	12.05	32.24
59	Chandauli	32.22	4.66	36.88	11.60	48.05
60	Varanasi	16.27	2.23	18.50	3.40	21.83
61	Sant Ravidas Nagar (Bhadohi)	49.19	8.43	57.61	11.57	68.51
62	Mirzapur	30.94	12.05	42.99	13.71	56.12
63	Sonbhadra	32.21	11.20	43.41	8.21	51.27
64	Etah	40.81	12.53	53.34	9.56	62.39
65	Kanshiram Nagar	56.88	11.83	68.71	24.04	91.10
66	Amethi	70.98	22.60	93.58	19.09	110.89
67	Budaun	39.04	21.66	60.70	5.31	65.69
68	Ghaziabad	35.76	5.35	41.10	0.00	41.10
69	Hapur	36.08	23.57	59.66	1.72	61.27
70	Moradabad	42.18	15.50	57.69	9.62	66.75
71	Muzaffarnagar	13.15	2.17	15.32	2.53	17.81
72	Rae Bareli	32.09	4.19	36.28	6.51	42.55
73	Sambhal	45.58	21.06	66.64	11.95	77.80
74	Shamli	38.20	16.01	54.22	7.04	60.88
75	Sultanpur	34.06	6.91	40.97	4.73	45.51
A	Aspirational districts*	32.75	13.53	46.28	10.70	56.48
B	Non-aspirational districts	36.04	14.78	50.82	9.90	60.22
	Uttar Pradesh	35.71	14.65	50.37	9.98	59.85

Neonatal mortality rate (NMR)

Estimates of neonatal mortality (deaths in the first 28 days per 1,000 live births) vary widely across UP's 75 districts. The state's average NMR is approximately 36 per 1,000 live births, but individual districts range from rates in the single digits up to the 60s. Notably, Amethi recorded the highest NMR at about 71 per 1,000, and several other districts in western and central UP (e.g., Mainpuri and Shahjahanpur) have NMR levels above 60. In contrast, a few eastern districts report dramatically lower NMRs: Ballia's NMR is under 10 per 1,000, the lowest in the state, and Deoria and Jaunpur are around 11-12 per 1,000. Even Siddharthnagar, an aspirational district in eastern UP, achieved an NMR of about 13.5, coming close to the SDG target of ≤12. These disparities suggest pockets of relative success in neonatal survival (particularly in parts of eastern UP) alongside areas with persistently high newborn mortality. Districts with the highest NMR tend to be those where health services and maternal-newborn care may be lagging, indicating that newborn survival is still a critical challenge in large swaths of the state. The extremely low NMRs reported in a few districts might partly reflect recent improvements or data variability, but they highlight that achieving near-SDG levels is possible in UP, given the right conditions. Overall, however, most districts remain far above the neonatal mortality target, underscoring the need for intensified focus on prenatal and immediate postnatal care in the high-mortality districts.

Post-neonatal mortality rate (PNMR)

Post-neonatal mortality estimates (deaths occurring from one month up to 12 months of age, per 1,000 live births) show a similarly broad range across UP's districts. The state average PNMR is about 14.7 per 1,000. A majority of districts have PNMRs in the low-to-mid teens, indicating that many infant deaths beyond the first month are being prevented. However, some districts still experience very high post-neonatal mortality, suggesting ongoing vulnerabilities to infant illnesses after the neonatal period. The highest PNMR is observed in Agra, at roughly 32.4 per 1,000, more than double the state average, which implies significant infant mortality in the later infancy stage (likely due to infections or other postnatal causes). A cluster of other districts, including Hardoi (26.6), Hapur (23.6), Kheri (25.3), and Amethi (22.6), also have PNMRs well above 20. By contrast, several districts report very low PNMRs, under five per 1,000. For example, Kushinagar's PNMR is only 1.9, and Muzaffarnagar and Varanasi are around 2.2. Such low post-neonatal rates rival levels seen in much more developed settings and suggest that in those districts, infant care and disease prevention in months 2-12 of life have been remarkably effective. The extreme gap between places like Kushinagar and Agra underscores uneven progress in some locales; immunization, nutrition, and management of infant illnesses (diarrhea, pneumonia, etc.) have greatly reduced post-neonatal deaths, whereas in others these measures need urgent strengthening. Many districts in eastern UP (e.g., Mau, Deoria, Jaunpur) also show PNMR in the single digits, reflecting better survival of infants after the neonatal period. Overall, while roughly half of UP's districts keep post-neonatal mortality below 10, a significant number still suffer rates above 20. This pattern indicates that preventable infant deaths (after the first month) remain a concern in specific high-PNMR districts, likely tied to disparities in healthcare access, infant feeding practices, and socioeconomic conditions.

Child mortality rate (CMR) (1-4 years)

The estimates of CMR (deaths at ages 1-4 years per 1,000 live births) in UP show a notably better overall picture, with the state average around 10 per 1,000. In fact, many districts have brought CMR down into single digits or even effectively zero, indicating that survival of children who reach their first birthday has improved substantially across the state. Nearly all districts report CMR below 17, and a considerable number are under five. For instance, Ghaziabad and Hamirpur reported zero child deaths (CMR 0) in the 2019-2021 period, suggesting that these areas saw virtually no deaths among children 1-4 years old in recent years. Similarly, Deoria, Azamgarh, and Basti have very low CMRs (around 2-6 per 1,000), meaning the vast majority of children who survive infancy in those districts live to age five. This represents significant progress, as child mortality (1-4 years) tends to fall with improvements in nutrition, immunization, and treatment of childhood illnesses. However, a few outlier districts still experience alarmingly high mortality in the 1-4-year age group. Fatehpur stands out with a CMR of about 29 per 1,000, nearly three times the state average and by far the highest in UP, signaling a persistent child health crisis there (perhaps due to malnutrition or poor healthcare for toddlers). Kanshiram Nagar (Kasganj) and Prayagraj (Allahabad) also have elevated CMRs around 24-25, well above most districts. A handful of others (e.g., Shravasti, Kheri, Pratapgarh) show CMRs in the mid-to-high teens, indicating room for improvement. Nonetheless, the overall pattern is that child (1-4) survival in UP is markedly better than infant survival and many districts have nearly eliminated deaths in this age range. The few districts with very high CMR are exceptions that require targeted interventions (such as nutrition programs or pediatric care services) to address whatever localized factors (disease outbreaks, malnutrition, etc.) are driving child mortality there. The near-zero CMR in some districts demonstrates what is achievable, as children who survive infancy can largely be kept alive with basic health interventions like vaccination, oral rehydration for diarrhea, and continued nutrition support.

Under-five mortality rate (U5MR)

The estimates of U5MR (probability of dying before age five per 1,000 live births) encapsulate the combined impact of infant and child mortality. UP's overall U5MR is about 60 per 1,000 for the 2019-2021 period, which means roughly 6% of children born in UP still do not survive to their fifth birthday. This state average is one of the highest in India, and district-level U5MR figures reveal enormous internal disparities. The worst-affected districts have under-five mortality comparable to some of the poorest regions in the world, whereas the best-performing districts are approaching levels seen in middle-income countries. Amethi has the highest U5MR in the state at approximately 111 per 1,000 live births, indicating that more than one in 10 children born in Amethi die before age five. Several other districts also show extremely high child mortality: Shahjahanpur (101), Mainpuri (95), Sitapur (96), Hardoi (93), and Kanshiram Nagar (91) all have U5MRs around 90-100 per 1,000. These figures reflect the compounding of both high infant deaths and notable child deaths in those areas, a serious concern for public health. By stark contrast, a number of districts have achieved U5MRs at or below the SDG threshold of 25 per 1,000. Jaunpur (17.4) and Muzaffarnagar (17.8) stand out with the lowest U5MRs in UP, followed closely by Deoria (18.7). Siddharthnagar (20.5) and Basti (33.8) also report greatly reduced under-five mortality relative to the state average. Indeed, it is striking that in a handful of eastern and western UP districts, only around 2% of children die before age five, a level on par with India's national average, whereas in the worst districts, this risk is six times higher. The geographic pattern is mixed: some eastern districts (like Ballia, Deoria, Jaunpur) have very low U5MR, but others in the east (like Mau at 56.9) remain high; similarly, while most of the highest U5MRs are in central/northwestern UP, a western district like Muzaffarnagar is among the lowest. On the whole, around two dozen UP districts report U5MR below 50, while roughly an equal number exceed 70. This broad divergence highlights that local factors, quality of healthcare services, maternal education, poverty levels, etc. play a major role. Districts that have succeeded in lowering under-five mortality likely benefit from better primary healthcare (e.g., immunization coverage and newborn care) and social determinants, whereas those still with very high U5MR clearly need intensified efforts to address the causes of death among newborns and young children. Importantly, in all districts, the bulk of under-five deaths occur in the first year of life; reducing overall U5MR will hinge on lowering neonatal and infant mortality in particular.

Focus on aspirational districts

The Government of India's "Aspirational Districts" initiative identified eight lagging districts in UP for focused development: Bahraich, Balrampur, Chandauli, Chitrakoot, Fatehpur, Shravasti, Siddharthnagar, and Sonbhadra. An analysis of the NFHS-5 data for estimating child mortality indices suggests that, taken as a group, these aspirational districts are performing slightly better than the state average on most child survival indicators. Their combined U5MR is 56.5 per 1,000, a bit lower than the overall UP U5MR of 59.9. The neonatal (0-1 month) and infant mortality rates in these districts averaged around 33 and 46 per 1,000, respectively, both marginally below the state averages (35.7 and 50.4). This is an encouraging sign that intensified interventions and resources in those districts may be yielding improvements. For example, Siddharthnagar, one of the aspirational districts, has among the lowest mortality rates in the state (NMR 13.5, U5MR 20.5), demonstrating remarkable progress. Chitrakoot and Chandauli also register U5MRs (around 49 and 48 per 1,000) that are significantly below the state average, alongside relatively low neonatal and child mortality, suggesting broad-based improvements in child health services. On the other hand, not all aspirational districts are uniformly outperforming the rest of UP. Bahraich and Balrampur, for instance, still have quite high infant and under-five mortality (Bahraich U5MR 69, Balrampur 65), higher than the state mean. And Fatehpur stands out negatively for its child mortality: with a CMR of 29, Fatehpur has one of the worst 1-4-year survival rates in the entire state, which skews the average CMR of the aspirational group slightly above the UP average (10.7 vs. 10.0). Excluding Fatehpur, most of the aspirational districts show child mortality indicators on par with or better than UP as a whole. The fact that, on aggregate, the aspirational districts have lower neonatal, post-neonatal, infant, and under-five mortality than non-aspirational districts suggests that targeted health and development programs in those areas (such as special funding for health facilities, nutrition missions, and intensive monitoring) are making an impact. In summary, these eight districts, historically among the most underdeveloped, now generally exhibit moderate improvements in child survival, though internal challenges remain (especially in Fatehpur and pockets of Bahraich/Balrampur). Continued focus on these districts is needed to sustain and accelerate the gains, ensuring that even the outliers within this group catch up to desired levels.

## Discussion

UP's CMRs, although improving, remain well above national averages and global targets. According to the NFHS 2019-2021, India's U5MR was about 42 per 1,000 (down from 50 in 2015-2016), and the neonatal mortality was about 25 per 1,000 [11]. UP's corresponding rates were roughly 60 and 36, respectively, in the same period [12]. In fact, the previous NFHS (2015-2016) had put UP's U5MR near 78 per 1,000, the highest among all states [13], so while the state has achieved a substantial reduction to 60, it continues to lag the country by a wide margin. This gap is corroborated by SRS data, as of 2020, UP's U5MR was still considerably higher than the national U5MR [14]. However, the eight Empowered Action Group (EAG) states and Assam continue to bear a disproportionately high burden of under-five deaths compared with southern and western non-EAG states, largely due to lower socioeconomic development, poorer health infrastructure, and limited access to essential services [15]. Some better-off Indian states (Kerala, Tamil Nadu, etc.) have already met the SDG on under-five mortality (≤25 per 1,000), whereas UP's average is more than double that target. The SDG aims for neonatal mortality not higher than 12 per 1,000 live births and under-five mortality below 25 by 2030 [16]. By these yardsticks, UP has a long journey ahead. Almost no UP district, except perhaps Ballia, currently meets the neonatal target, and only a handful (roughly 5-6 districts) are at or below the under-five goal. If current rates of decline continue, UP is unlikely to hit the 2030 targets, meaning an acceleration in progress is required.

On a positive note, the state did achieve noteworthy improvements between 2015 and 2021 (a roughly 25% reduction in under-five mortality). Additionally, UP showed the largest recent decline in U5MR among states from 2019 to 2020 (a drop of 5 points in one year) [17], indicating that intensified child health efforts can yield rapid gains. Another encouraging sign is the near-eradication of deaths in the 1-4-year age group in many districts, an aspect where UP is now close to national performance. This means the infant period (especially the first month of life) accounts for an overwhelming share of child mortality in UP, as in India overall. Nationally, about 48% of under-five deaths now occur in the first week of life and 74% in the first year. Significant progress has been made in reducing under-five mortality in India over the past three decades, yet stark regional and socioeconomic disparities persist. Between 1993 and 2021, India's national U5MR declined substantially, driven largely by sharp reductions in post-neonatal and child mortality, although early-neonatal mortality showed relatively slower improvement [18]. Data from the NFHS-5 (2019-2021) compared with NFHS-4 confirm continued declines in infant and under-five mortality across most states, accompanied by improvements in coverage of key maternal and child health interventions [12]. UP likely mirrors this pattern, implying that further mortality reduction hinges on improvements in maternal-newborn care and infant health. In summary, UP has made progress (closing some of the gap from NFHS-4 to NFHS-5), but it remains an outlier with CMRs far above India's average and nowhere near the SDG targets. Continued monitoring against these benchmarks, and learning from better-performing states, will be crucial as UP works to catch up.

The stark intra-state differences and the gap with national goals carry important policy implications. First, UP must prioritize interventions in high-mortality districts. The data show that certain districts (like those in central UP with high NMR/U5MR and outliers such as Fatehpur) are pulling the state averages up. Targeted programs focusing on these worst-affected areas, similar to the Aspirational District model, could help replicate the successes seen in places like Siddharthnagar. Key interventions should center on the neonatal period, since newborn deaths are the largest component of under-five mortality. This calls for strengthening maternal health and delivery care: increasing institutional (hospital) deliveries with skilled birth attendants and ensuring the presence of emergency obstetric and newborn care in each district. It is notable that nearly 40% of maternal and neonatal deaths in India occur during delivery or within the first 24 hours after birth and UP's high NMR reflects this critical window. Expanding schemes like the Janani Suraksha Yojana (which promotes institutional delivery) and ensuring that every newborn is examined by a health professional within the first day or two of life can directly save lives. Indeed, nationally, the proportion of newborns receiving a check-up within two days jumped from 27% to 82% between 2015 and 2021, a change credited with helping reduce neonatal deaths. UP must ensure this postnatal care coverage is uniformly high across all districts.

Another priority is combating the causes of post-neonatal and child mortality, such as infections and malnutrition. The data suggest that where post-neonatal mortality remains high (e.g., Agra, Hardoi), broader healthcare access for infants is lacking. Interventions like universal immunization (Mission Indradhanush), management of childhood illnesses (Integrated Management of Neonatal and Childhood Illnesses guidelines), and improved infant feeding practices need to be bolstered in those districts. Malnutrition is an underlying factor that still plagues UP, as per NFHS-5, 40% of under-fives in the state are stunted. This contributes to child mortality by weakening immunity. Nutrition programs (including breastfeeding promotion, supplementation, and food security schemes) are thus vital parts of a child survival strategy. The state's ongoing POSHAN Abhiyaan and supplementary feeding through Anganwadi centers must reach the most vulnerable communities to reduce malnutrition-related deaths.

The extremely low child mortality in some districts shows that existing government programs can work when effectively implemented. For instance, the Home-Based Newborn Care (HBNC) program sends ASHA health workers for multiple home visits in an infant's first 42 days of life, providing screening and counseling. Where ASHAs are active and well-supported, newborn survival improves. However, gaps remain: many families, especially in remote or underserved areas, do not fully benefit from such schemes. UP has a high proportion of home births and home-based neonatal care, especially in rural pockets. The lack of trained birth attendants and poor hygiene in home deliveries dramatically increase infection risks for newborns. Thus, the state should intensify training and deployment of skilled personnel (doctors, nurses, midwives) in rural health facilities and promote clean delivery practices. Ensuring ambulance services and referral transport (through initiatives like the Janani Shishu Suraksha Karyakram (JSSK), which guarantees free maternity and newborn services including transport) are functional in every district will encourage timely care-seeking. Notably, while schemes like JSSK and HBNC are in place, their coverage is uneven; audits have found that not all eligible families receive the free services and home visits intended. Strengthening oversight and accountability in these programs is needed so that their benefits uniformly reach high-risk populations, such as poor families, remote villages, and marginalized groups with historically higher child mortality.

UP's government has recently reported improvements, citing declines in U5MR and improvements in institutional delivery and immunization. The emphasis on inter-sectoral initiatives, improving water, sanitation, female literacy, and poverty alleviation, also cannot be overstated, as these factors closely tie into child survival. Child mortality is a multifaceted problem: for example, better female education and empowerment can lead to improved child care practices and health service utilization, ultimately lowering mortality. Thus, policies under the NHM should dovetail with missions on rural development, women's education, and social welfare in UP. Given the data, special attention should also be paid to maternal health and nutrition, since healthy mothers are less likely to have low-birth-weight babies who are at higher risk of neonatal death. UP's maternal mortality and child mortality are linked issues; strengthening antenatal care, nutrition for pregnant women, and preventing teenage pregnancies will improve newborn outcomes, too.

Finally, greater data-driven management is implied: the fact that districts like Ballia and Deoria have achieved very low mortality suggests there are best practices or community factors there that could be studied and replicated. Conversely, high-mortality districts should receive focused audits, e.g., death reviews to understand every neonatal and child death's cause and whether it was preventable. This aligns with India's RMNCH+A (Reproductive, Maternal, Newborn, Child, Adolescent Health) strategy of using data to pinpoint gaps. In summary, achieving the child mortality reductions needed in UP will require a comprehensive approach: strengthening healthcare systems (especially perinatal and infant care), improving nutrition and public health measures, and addressing the socioeconomic determinants in the poorest communities. Without such concerted efforts, UP will remain off-track for the SDG 3.2 goals; with them, however, the state could accelerate its recent progress and save tens of thousands of young lives each year.

## Conclusions

UP's latest district-level data on child mortality (NFHS-5, 2019-2021) reveal both hopeful trends and persistent challenges. All four key indicators, namely, NMR, PNMR, CMR, and U5MR, have improved compared to previous years, and a few districts have attained mortality levels on par with national averages or SDG benchmarks. Yet the overall under-five mortality in UP remains distressingly high, with huge disparities across districts. Most child deaths are now concentrated in the neonatal period, underscoring the need for urgent improvements in maternal and newborn health services. Strategic, evidence-based interventions, particularly targeted at the highest-mortality districts and aimed at the first days and months of life, will be critical for UP to bridge the gap with the rest of India. In sum, while progress is visible and some districts offer success stories, child survival in UP still demands focused policy priority. By strengthening healthcare delivery and addressing root causes like infections, malnutrition, and inadequate prenatal care, the state can move closer to the goal of ensuring that every child born in UP has an equal chance of reaching their fifth birthday.
